# Pushing periodic-disorder-induced phase matching into the deep-ultraviolet spectral region: theory and demonstration

**DOI:** 10.1038/s41377-020-0281-4

**Published:** 2020-03-18

**Authors:** Mingchuan Shao, Fei Liang, Haohai Yu, Huaijin Zhang

**Affiliations:** State Key Laboratory of Crystal Materials and Institute of Crystal Materials, Shandong University, Jinan, 250100 China

**Keywords:** Applied optics, Lasers, LEDs and light sources

## Abstract

Nonlinear frequency conversion is a ubiquitous technique that is used to obtain broad-range lasers and supercontinuum coherent sources. The phase-matching condition (momentum conservation relation) is the key criterion but a challenging bottleneck in highly efficient conversion. Birefringent phase matching (BPM) and quasi-phase matching (QPM) are two feasible routes but are strongly limited in natural anisotropic crystals or ferroelectric crystals. Therefore, it is in urgent demand for a general technique that can compensate for the phase mismatching in universal nonlinear materials and in broad wavelength ranges. Here, an additional periodic phase (APP) from order/disorder alignment is proposed to meet the phase-matching condition in arbitrary nonlinear crystals and demonstrated from the visible region to the deep-ultraviolet region (e.g., LiNbO_3_ and quartz). Remarkably, pioneering 177.3-nm coherent output is first obtained in commercial quartz crystal with an unprecedented conversion efficiency above 1‰. This study not only opens a new roadmap to resuscitate those long-neglected nonlinear optical crystals for wavelength extension, but also may revolutionize next-generation nonlinear photonics and their further applications.

Dear Editor,

In 1961, nonlinear second-harmonic generation (SHG) was first discovered in quartz crystal^1^. In nonlinear parametric process, the phase-matching condition (the momentum relation between the fundamental and harmonic light) is indeed the most critical, corresponding to a constructive interference enhancement in a nonlinear medium and high-efficiency generation proportional to the crystal length^2^. Currently, associated with the natural birefringence^3^ of nonlinear crystals at typical angles (Fig. 1a) and periodic/aperiodic poled ferroelectric domains^4,5^ (+***P*** and −***P***) (Fig. 1b) in certain nonlinear crystals^6–8^, coherent generation ranging from the visible region to the terahertz region has been developed and utilized in classical and quantum regions^9–11^. However, most nonlinear optical crystals have neither sufficient birefringence nor controllable ferroelectric domains. Therefore, it is in urgent demand for the development of a new route to achieve phase matching in arbitrary nonlinear crystals and in broad wavelength ranges.

**Fig. 1:**
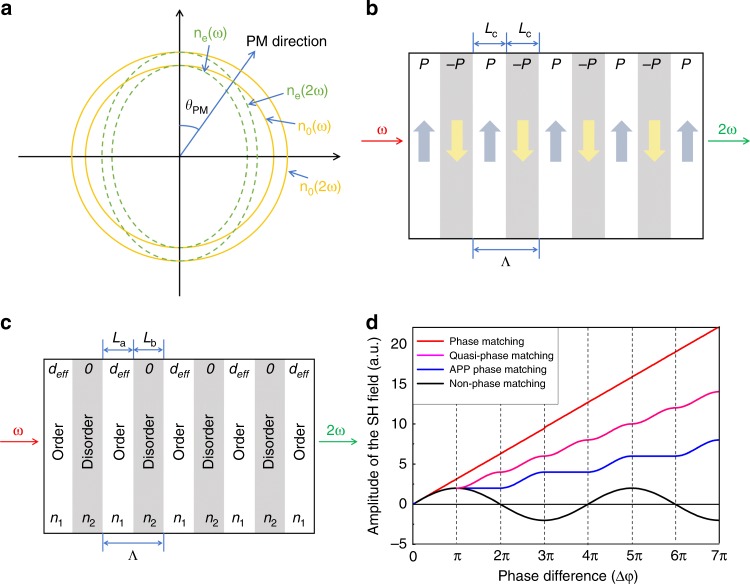
APP phase matching for nonlinear frequency conversion. **a** Schematic graph of the birefringent phase-matching condition in a negative uniaxial crystal. *θ*_PM_ is the phase-matching angle. Along this direction, *n*_*o*_(*ω*) = *n*_*e*_(2*ω*) and Δ*k* = 0. **b** Schematic graph of the quasi-phase-matching condition in a ferroelectric crystal. The up and down arrows represent positive and negative ***P*** polarizations, respectively. The period length Λ is double the coherence length *L*_c_ (Λ = *L*_c_ + *L*_c_). **c** Schematic graph of the additional phase-matching condition in arbitrary nonlinear optical crystals. The white and gray regions represent the ordered crystal and the disordered amorphous state, respectively. The period length Λ equals the sum of the ordered width *L*_a_ and disordered width *L*_b_ (Λ = *L*_*a*_ + *L*_*b*_). Notably, *L*_a_ and *L*_b_ may be equivalent to *L*_c_ or an integer multiple of *L*_c_. *d*_eff_/0 and *n*_1_/*n*_2_ represent the second-order nonlinear coefficient and refractive index of the ordered and disordered regions, respectively. **d** The amplitude of the generated second-harmonic (SH) field under phase-mismatching and different phase-matching conditions. Equal interaction lengths of the nonlinear media and the same efficient nonlinear coefficients *d*_eff_ are assumed. The period length Λ for quasi-phase-matching and additional phase matching is 2*L*_c_ (Λ = *L*_c_ + *L*_c_)

Deep-ultraviolet (DUV) coherent light is a fundamental source for an angle-resolved photoemission system^12^, photolithographic techniques^13^, and Raman spectroscopy^14^. The generation of DUV light by nonlinear frequency conversion is considered to be the “Holy Grail” in nonlinear optics^15^. To the best of our knowledge, only potassium beryllium fluoroborate KBe_2_BO_3_F_2_ (KBBF) nonlinear crystal has achieved direct birefringent phase-matchable SHG in the DUV region^16^. However, the wide application of KBBF crystals is limited by the great difficulty of crystal growth and toxicity of the raw material BeO^17^. In addition, random quasi-phase matching has also been realized in SrB_4_O_7_ crystal using a spontaneous domain structure^18^. However, the SHG conversion efficiency is too low (only 0.04‰) to be widely applied.

Herein, we propose an original concept, the additional periodic phase (APP) from order/disorder alignment (Fig. 1c), which can compensate for the mismatched phase in arbitrary nonlinear optical crystals. Moreover, the efficient SHG output in the visible, ultraviolet and even deep-ultraviolet regions is experimentally demonstrated in LiNbO_3_ and quartz. Remarkably, 177.3-nm coherent output is obtained in a quartz crystal with an unprecedented conversion efficiency above 1‰.

Taking the typical collinear frequency-doubling as an example, the electric field *E*_*2ω*_(*z*) of SHG light is described as^2^:

1 $$\frac{dE_{2{\omega}}(z)}{dz} =\frac{2i{\omega}}{cn_{2{\omega}}(z)}d_{{\rm eff}}(z)E_{\omega}^{2}e^{-i{\it{\Delta}}{\varphi}}$$

where *E*_*ω*_ (*z*) denotes the electric field of the fundamental field at the propagation length *z*; *ω* refers to the fundamental frequency; *c* represents the light velocity; *n*_*2ω*_ (*z*) and *d*_eff_ (*z*) denote the refractive indices of the SHG light and effective nonlinear coefficient at the propagation length *z*, respectively; and ${\mathrm{\Delta }}\varphi = \Delta kz = \left( {k_2 - 2k_1} \right)z$ is the phase difference between the fundamental and SHG lights with the wavevectors *k*_1_ and *k*_2_, respectively.

Under the phase-mismatching condition, the amplitude of the SHG electric field will oscillate with a phase difference of 2*π* in one period (black line in Fig. 1d). There is no effective coherent output under the phase-mismatching condition except for a weak SHG signal. Assuming that the phase-matching condition is perfectly satisfied, the SH field amplitude consequently grows linearly with the phase difference (red line in Fig. 1d). In a ferroelectric crystal, quasi-phase matching can be achieved by adding the reciprocal vector (*G*) with periodic reversal polarization (the sign of *d*_eff_) to satisfy $\Delta k_z = k_z^2 - 2k_z^1 - mG = 0$, where *m* is an integer. Consequently, the generated SH field can continuously increase along the propagation direction (pink line in Fig. 1d). Compared with birefringent phase matching, quasi-phase matching is not limited by the special direction so as to use the largest *d*_*eff*_ in certain nonlinear crystals, e.g., PPLN and PPKTP.

In accordance with the theory of quasi-phase matching, an additional periodic phase (APP) Δ*φ*_APP_, resulting from the alignment of order/disorder (crystal/amorphous) species, is proposed to compensate for the phase difference Δ*φ*_PD_ between the fundamental and SHG lights. The APP concept means that after the light propagates over the coherence length *L*_c_, the generated phase difference Δ*φ*_PD_ is compensated by the additional phase difference Δ*φ*_APP_ with Δ*φ*_APP _+ Δ*φ*_PD_ = 0 or 2*mπ* (*m* is an integer). The APP can be realized by periodically engineering regions in nonlinear crystals to undermine the translational symmetry of the nonlinear crystal and block the conversion of the energy from the SHG to the fundamental light, in addition to the oscillation of the SHG electric amplitude (blue line in Fig. 1d). The detailed mathematical analysis about the APP concept is presented in the Supplementary Information. In non-phase-matching condition, the APP of Δ*φ*_APP_ = (2*m* + 1)*π* and Δ*φ*_APP_ = 2*mπ* will introduce constructive and destructive contributions to the SHG signal, respectively. Nevertheless, in our proposed APP strategy, the positive contribution from Δ*φ*_APP_ = (2*m* + 1)*π* remains, but the contribution from Δ*φ*_*APP*_ = 2*mπ* decreases to zero, not a negative value. We can attribute the APP to the periodic variation of refractive index (*n*_1_ in the crystalline region and *n*_2_ in the amorphous region) and effective nonlinear coefficient *d*_*ij*_ (*d*_eff_ in the crystalline region and zero in amorphous region). The periodic length Λ is the sum of the coherent length *L*_a_ and the coherent length *L*_b_. Accordingly, an additional reciprocal vector $G = \frac{{2\pi }}{\Lambda }$ is introduced. When *G* = Δ*k*, the APP phase matching can achieve efficient SHG output, revealing that we can assume the proposed APP phase matching to be a new type of quasi-phase matching. In contrast to traditional quasi-phase matching based on the reversal domains in a few ferroelectric crystals, the proposed APP phase matching exhibits remarkable advantages in relaxing the phase-matching requirements and utilizing the largest efficient nonlinear coefficient *d*_eff_. Accordingly, this new APP phase-matching concept should be applicable to all non-centrosymmetric crystals to achieve highly efficient SHG output, even crystals without sufficient birefringence or reversible ferroelectric domains.

To experimentally demonstrate APP phase matching in nonlinear optical crystals, techniques to fabricate photonic crystals (e.g., laser writing processing and ion beam etching) were utilized^19^, which can easily undermine the translational symmetry of a nonlinear crystal and generate amorphous regions in the nonlinear crystal, as shown in Fig. 1c. In this scenario, if the propagation length *L*_b_ of the light in the amorphous regions is controlled, the phase difference Δ*φ*_PD_ generated in the crystalline region can be compensated according to the introduction of the APP with Δ*φ*_APP_ + Δ*φ*_PD_ = 0 or 2*mπ*, only when Δ*φ*_PD _≠ 2*π*.

First, a 1064-nm SHG experiment was performed in both ferroelectric LiNbO_3_ and non-ferroelectric quartz. A Nd:YAG laser (100 ns, 20 kHz) illuminated an as-prepared sample by a focusing lens (*f* = 100 mm), and the SHG power was collected by a power meter (Fig. 2a). In previous studies, LiNbO_3_ and quartz are two common media used for optical waveguides in integrated photonics. With a suitable writing energy, amorphous LiNbO_3_ and quartz regions could be produced with a tunable period. A 350-fs pulsed laser at 1040 nm was used for the lasing writing. The LiNbO_3_ and quartz crystals were cut along the X/Z direction to use the largest SHG coefficient *d*_33_/*d*_11_ in the nonlinear optical process. The periodic lengths Λ of LiNbO_3_ and quartz are 6.79 μm and 41.6 μm for 1064-nm SHG conversion, respectively. By studying the grating diffraction of the samples based on Bragg diffraction (Supplementary Fig. S1), the refractive index divergence (Δ*n* = *n*_1_ – *n*_2_) between crystalline and amorphous regions is very small, only 0.003–0.004 in LiNbO_3_ and 0.004–0.005 in quartz from 350 to 630 nm (Fig. 2b). This is consistent with the reported conclusions in laser-written LiNbO_3_^20^ and SiO_2_ waveguides^19^, suggesting that laser writing would not greatly break the dispersion of the refractive index. In addition, the broken translational symmetry in the written regions was also studied using the conoscopic interference technique (Supplementary Fig. S3). The results reveal that no obvious birefringence emerged along the X direction in the amorphous regions, indicating isotropic propagation of the fundamental and SHG waves. We have also demonstrated the SHG signal of the APP SiO_2_ sample with a second-harmonic microscope. The beam with a wavelength of 1030 nm is focused inside the quartz crystal through an objective lens (Fig. 2c). The upper part of the crystal is unprocessed, while the lower part is a processed periodic grating. There is no SHG signal for phase mismatching in the unprocessed region. In contrast, we can see a clear SHG signal in the region of the periodic grating, which proves the feasibility of the APP theory.

**Fig. 2:**
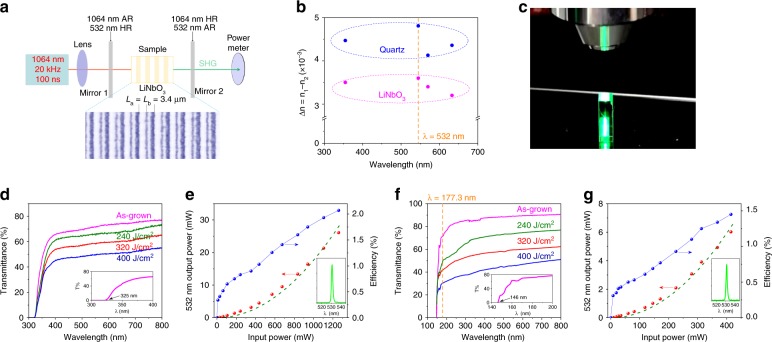
Experimental demonstration of the APP SHG process for 1064 nm laser. **a** Experimental setup used in the SHG experiment. The blue map is a micrograph of the as-prepared LiNbO_3_ sample with *L*_a_ *=* *L*_b_ = 3.4 μm. **b** The refractive index fluctuation of the LiNbO_3_ (pink circle) and quartz (blue circle) samples in the amorphous region. **c** Experimental demonstration of the SHG signal of the APP SiO_2_ sample. **d** Transmission spectra of the unprocessed and laser-written LiNbO_3_ samples for different writing energy intensities (the green, red, and blue lines correspond to samples written by laser energy intensities of 240, 320, and 400 J/cm^2^, respectively). The inset is an enlarged transmittance spectrum of the as-grown LiNbO_3_ crystal. **e** 532-nm SHG output power in LiNbO_3_ (red point) and the optical conversion efficiency (blue point) with Λ = 6.79 μm. The green dotted line is the quadratic fitting curve for the SHG output power with an R factor equal to 0.986. The inset is a spectral graph of the SHG signal at 532 nm. **f** Transmission spectra of the unprocessed and laser-written quartz samples for different writing energy intensities (the green, red, and blue lines correspond to samples written by laser energy intensities of 240, 320 and 400 J/cm^2^, respectively). The inset is an enlarged transmittance spectrum of the as-grown quartz crystal. **g** 532-nm SHG output power in quartz (red point) and the optical conversion efficiency (blue point) with Λ = 41.6 μm. The green dotted line is the quadratic fitting curve for the SHG output power with an R factor equal to 0.991. The inset is a spectral graph of the SHG signal at 532 nm

Figure 2d shows the transmission spectra of the as-grown crystals and APP LiNbO_3_ samples processed with different writing energy intensities. The result suggests that the transparent cutoff edges of the prepared LiNbO_3_ samples are almost unchanged at 325 nm. However, their transmission gradually reduces with increased writing energy intensity and becomes lower than 50% under an energy intensity of 400 J/cm^2^. Using APP LiNbO_3_ with Λ = 6.79 μm and Δ*φ*_APP_ = Δ*φ*_PD_ = *π* at the fundamental wavelength of 1064 nm, the frequency-doubling performance at 532 nm (insert graph in Fig. 2e) was studied. The significant SHG output power *P*_2*ω*_ is 26.11 mW at 532 nm for a pump power *P*_0_ of 1260 mW (Fig. 2e). The optical conversion efficiency *η* (*η* = *P*_2*ω*_/*P*_0_) is 2.07%. Meanwhile, quartz samples exhibit a shorter transmittance edge of 146 nm and a gradually reduced transmission with an increased writing energy intensity (Fig. 2f). The frequency-doubling signal at 532 nm is also detected in APP quartz (insert graph in Fig. 2g). The preliminary SHG output power *P*_2*ω*_ is 6.02 mW at 532 nm for a pump power *P*_0_ of 415 mW (Fig. 2g). Notably, as a non-birefringent-matched nonlinear optical crystal, the conversion efficiency *η* in APP quartz is calculated to be 1.45%, which is approximately two orders of magnitude higher than the recently reported SHG result in a well-designed silica microcavity (~0.049%)^21^ and in periodic stacking quartz plates (~0.01%) via traditional quasi-phase matching^22^. This result strongly demonstrates that our proposed APP phase-matching strategy is indeed effective.

Next, we demonstrated the APP strategy in the ultraviolet and solar-blind regions. Limited by the redshifted cutoff edge of LiNbO_3_, we only used quartz as an example. Two different periodically broken regions were designed in APP quartz with lengths of *L*_a_ = *L*_b_ = 2.1 μm (sample A) and 1.4 μm (sample B), corresponding to an APP of *Δφ*_APP_ and a phase difference of *Δφ*_PD_ Δ*φ*_APP_ = Δ*φ*_PD_ = *π* at SHG wavelengths of 242 nm and 214 nm, respectively. Meanwhile, samples A and B are also relative to Δ*φ*_APP_ = Δ*φ*_PD_ = 3*π* and 2*π* at the SHG wavelength of 177.3 nm (Fig. 3a), respectively. As mentioned above, the optical conversion efficiency strongly depends on the fluctuation of the phase difference. Only Δ*φ*_APP_ = Δ*φ*_PD_ = (2*m* + 1)*π* is required to satisfy the phase-matching criteria, but Δ*φ*_APP_ = Δ*φ*_PD_ = 2*mπ* still maintains the non-phase-matching condition. Remarkably, Δ*φ*_APP_ = Δ*φ*_PD_ = *π* exhibits the highest SHG conversion efficiency for the same crystal length (Fig. 3b). An optical parametric oscillation laser with a pulse width of 10 ns was employed in the ultraviolet SHG experiments. The fundamental source ranges from 410 nm to 2200 nm. As shown in Fig. 3c, e, the broadband phase-matching condition was achieved in both samples A and B owing to the aperiodic broken regions and fluctuated reciprocal vectors (Supplementary Figs. S4, S5). However, the strongest output signal is centered at 242 nm and 214 nm in samples A and B, respectively, which is consistent with our theory. In sample A, the output power increases with the square of the fundamental wave power, in which the maximum SHG output power $P_{2\omega }^A$ is 38.5 μW for an incident power *P*_0_ of 3.1 mW (Fig. 3d), relating to a high conversion efficiency *η* of 1.24%. In addition, in sample B, the output power also depends on the square of the input power of 428 nm. The highest $P_{2\omega }^B$ is 16.9 μW at a wavelength of 214 nm for an incident power *P*_0_ of 3.1 mW (Fig. 3f), corresponding to *η* of 0.55%. The slightly reduced efficiency in sample B can be attributed to a lower transmission and larger refractive indices with blueshifted wavelengths. These highly efficient results illuminate that our proposed APP strategy is also valid in the ultraviolet region.

**Fig. 3:**
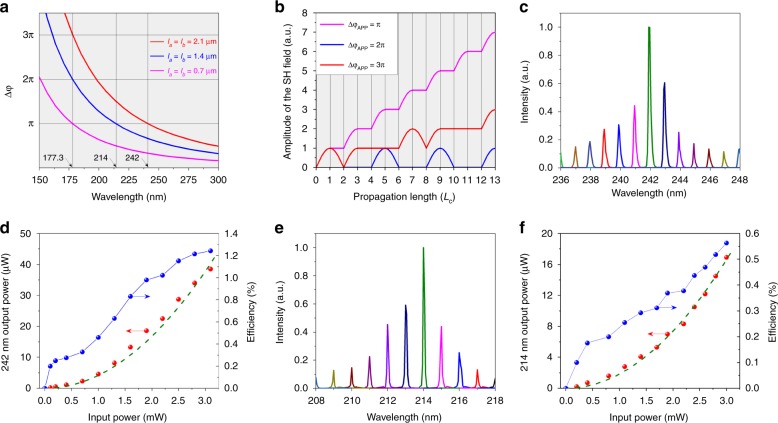
Experimental demonstration of the APP SHG process in the ultraviolet region. **a** Theoretical calculation of the APP (Δ*φ*_APP_) dispersion of the APP quartz samples with *L*_a_ = *L*_b_ = 2.1 μm, 1.4 μm, and 0.7 μm. **b** Schematic estimation of the SH field amplitude of the APP quartz with different shifted phases (Δ*φ*_APP_) for the same crystal length with coherent length *L*_*c*_. **c** SHG response at various fundamental wavelengths (236–248 nm) in APP quartz with *L*_a_ = *L*_b_ = 2.1 μm. **d** 242-nm SHG output power in quartz (red point) and the optical conversion efficiency (blue point) with *L*_a_ = *L*_b_ = 2.1 μm. The green dotted line is the quadratic fitting curve for the SHG output power with an R factor equal to 0.971. **e** SHG response at various fundamental wavelengths (208–218 nm) in APP quartz with *L*_a_ = *L*_b_ = 1.4 μm. **f** 214-nm SHG output power in quartz (red point) and the optical conversion efficiency (blue point) with *L*_a_ = *L*_b_ = 1.4 μm. The green dotted line is the quadratic fitting curve for the SHG output power with an R factor equal to 0.984

Finally, we attempted to extend the APP strategy into the deep-ultraviolet (DUV) region, an indispensable spectral range for high-resolution photolithography and angular resolution photoelectron spectrometry (ARPES)^23^. In particular, 177.3-nm coherent sources have greatly put forward electron state studies in strong correlation systems, such as topological insulators^24^, high-temperature superconductors^12^ and topological semimetals^25^. However, it is indeed difficult to achieve birefringent phase matching in the DUV region owing to the large requisite birefringence (at least 0.07). To date, direct phase-matchable SHG output has only been realized in KBBF crystal^16^. However, the strong layer growth habit and toxic BeO hinder its further applications in DUV coherent lasers. Despite many reported novel nonlinear optical materials with short absorption cutoffs in the deep-ultraviolet region, these materials could not generate deep-ultraviolet coherent light due to their insufficient birefringence^17^. Therefore, as a “Holy Grail” in the optical community, a 177.3-nm coherent laser still needs to be produced via other technical routes, just like APP.

First, we compared the basic properties of nine DUV transparent nonlinear optical crystals that have a short ultraviolet transparent cutoff wavelength^18,26–30^ to meet the primary requirement for APP crystals, as depicted in Fig. 4a. Among the crystals, only KBBF has demonstrated efficient deep-ultraviolet second-harmonic generation with birefringent phase matching^15,16^. By the comprehensive comparison shown in Supplementary Table S1, it can be found that the quartz and BPO_4_ should be optimized crystals for demonstrating APP 177.3-nm output in the DUV region. The calculated coherent lengths of these two crystals for the 355-nm SHG process are 0.701 and 0.785 μm^30^, respectively. However, it is still difficult to achieve a large BPO_4_ crystal with a high-optical quality due to its ultrahigh melting viscosity^31^. Therefore, commercial quartz was selected as the SHG crystal in the present DUV APP experiments. Quartz crystal has a maximum nonlinear coefficient of *d*_11_ = 0.3 pm/V, comparable to the nonlinear coefficient of KBBF (*d*_11_ = 0.47 pm/V)^15^. The birefringence (*n*_e _– *n*_o_) of quartz is only 0.01 at 355 nm, leading to impossible birefringent phase-matching^32^. In addition, quartz belongs to the nonpolar trigonal 32 system (*P*3_1_21 space group, No. 152), indicating that it has no reversal ferroelectric domains to achieve traditional quasi-phase matching. Second, the experimental setup of DUV SHG is also different from that of the early stated visible SHG process (Fig. 4b). Generally, 177.3-nm light will be strongly absorbed by oxygen in air. Therefore, the APP quartz and power meter were placed in a chamber full of nitrogen to eliminate the absorption of oxygen. An additional CaF_2_ prism was added to deflect and separate the fundamental (355 nm) and SHG (177.3 nm) signals. Third, as stated in Fig. 3a, the required period length *L*_a_ (and *L*_b_) for Δ*φ*_APP_ = Δ*φ*_PD_ = *π* of quartz is 0.7 μm. However, this value is smaller than the minimum accuracy of femtosecond laser manufacturing, as shown in Supplementary Fig. S4. Therefore, APP quartz with *L*_a_ = *L*_b_ = 2.1 μm was utilized to generate a 177.3-nm SHG laser, corresponding to a phase difference of Δ*φ*_APP_ = Δ*φ*_PD_ = 3*π*. Evidently, this conversion efficiency is lower than that the case for a phase difference of *π*. The quartz samples written with an energy intensity of 240J/cm^2^ were used to maintain a high DUV transmittance.

**Fig. 4:**
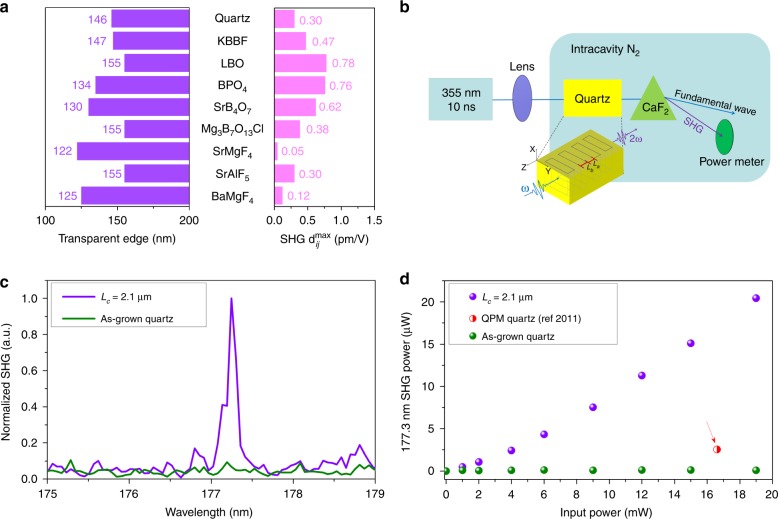
Experimental demonstration of the APP SHG process in the deep-ultraviolet region. **a** Comparison of common deep-ultraviolet transparent nonlinear optical crystals (only crystals with a transparent cutoff edge below 160 nm are plotted). **b** Experimental setup of the deep-ultraviolet SHG process from 355 nm to 177.3 nm. The focal length of the lens is 100 mm. The quartz sample and power meter were placed in a closed chamber filled with pure N_2_ to avoid SHG 177.3-nm absorption induced by oxygen. A CaF_2_ prism was employed to separate the fundamental and SHG signals. **c** SHG response signal (177.3 nm) in APP quartz with *L*_a_ = *L*_b_ = 2.1 μm (purple solid line) and as-grown quartz (green solid line). **d** 177.3-nm SHG output power in APP quartz (purple point) with *L*_a_ = *L*_b_ = 2.1 μm and *Δφ* = 3*π* and as-grown quartz (green point). The 193-nm output in quasi-phase-matched twined quartz (red point) is adapted from ref. ^33^

As depicted in Fig. 4c, an anticipated peak located at 177.3 nm emerges in APP quartz, which represents the first efficient DUV SHG output in quartz. In comparison, there is no detectable SHG signal in the as-grown crystalline quartz, suggesting that our phase-compensating strategy is indeed significant. With an improvement of the incident power *P*_0_ up to 19 mW, the output SHG power *P*_2*ω*_ increases to 20.4 μW, corresponding to an optical conversion efficiency *η* of 1.07‰ and a normalized conversion efficiency of *η*_*N*_ = 1.25 × 10^−5%^/W/cm^2^. As shown in Fig. 4d, the optical conversion efficiency of the present APP quartz is much higher than that of stressed twin quartz (*η* = 0.38‰, *η*_*N*_ = 2.18 × 10^−3%^/W/cm^2^) at the longer wavelength of 193 nm at the expense of the normalized conversion efficiency^33^. Theoretically, the SHG power should depend on the fundamental power quadratically. The near-linear relationship could be attributed to the additional influence of the beam size, wavelength, absorption, etc. Compared with the results in the visible and ultraviolet ranges, the relatively low conversion efficiency at 177.3 nm results from the inevitable absorption of the samples and residual oxygen, the loss on the ordered/disordered interface and the large APP of Δ*φ*_APP_ = 3*π*. The output power and conversion efficiency could be further enhanced by reducing the interface loss and realizing Δ*φ*_APP_ = Δ*φ*_PD_ = *π* in APP quartz with precise laser direct writing technology. Accordingly, the APP method should be a significant route to obtain deep-ultraviolet coherent lasers.

In summary, for the first time, a universal additional periodic phase for the phase-matching condition in nonlinear optics has been theoretically proposed, beyond traditional phase matching with natural birefringence of crystals and quasi-phase matching with reversible ferroelectric domains. APP technology is suitable for any acentric crystal (nonpolar, polar, and ferroelectric phases), and among such crystals, a nonpolar nonlinear crystal is the best candidate to demonstrate this theory. Taking LiNbO_3_ and quartz crystals as examples, an efficient 532-nm laser in the visible region and a 242/214-nm laser in the ultraviolet region were demonstrated by APP SHG conversion. In particular, deep-ultraviolet 177.3-nm generation was first achieved via a periodic disordered quartz crystal (nonpolar phase) with a high efficiency of 1.07‰. This APP strategy provides a versatile route for arbitrary nonlinear crystals at broadband wavelengths. Alongside the present SHG in the DUV region, it can be proposed that the APP strategy should also be available for a nonlinear optical process in the infrared region by using SHG, optical parametric oscillation, frequency difference, etc., where the phase difference Δ*φ*_PD_ could be compensated by the additional phase difference Δ*φ*_APP_. More importantly, this order/disorder alignment adds a variable physical parameter into the optical system, thus leading to a next-generation revolution in nonlinear or linear modulation and classical or quantum photonics.

## Supplementary information

Supplementary Information for Pushing periodic-disorder-induced phase matching into the deep-ultraviolet spectral region: theory and demonstration
