# Supplementary material for: Pushing periodic-disorder-induced phase matching into the deep-ultraviolet spectral region: theory and demonstration
Source: Light Sci Appl. 2020 Mar 18;9:45. doi: 10.1038/s41377-020-0281-4 (PMC7078200; doi:10.1038/s41377-020-0281-4)
Supplement: Supplementary file 1 — Supplementary Information for Pushing periodic-disorder-induced phase matching into the deep-ultraviolet spectral region: theory and demonstration [file 41377_2020_281_MOESM1_ESM.docx]

Supplementary Information

Pushing periodic-disorder-induced phase matching into the deep-ultraviolet spectral region: theory and demonstration

Mingchuan Shao, Fei Liang, Haohai Yu*, Huaijin Zhang**

State Key Laboratory of Crystal Materials and Institute of Crystal Materials, Shandong University, Jinan 250100, China.

*Corresponding author: E-mail: haohaiyu@sdu.edu.cn

**Corresponding author: E-mail: huaijinzhang@sdu.edu.cn

1. **Methods**

**Femtosecond laser writing of the APP SiO_2_ samples**

The LiNbO_3_ and quartz crystal was cut along the X/Z direction with the length of 5 mm to use the largest SHG coefficient *d_33_*/*d_11_* in nonlinear optical process. The infrared writing optical beam originated from a femtosecond laser (Spirit one 1040-8) operating at 1040 nm with a pulse duration of 350 fs and a repetition rate of 200 kHz. The samples were put on a stage which can move in three orthogonal directions with a resolution of 200 nm. The laser beam passed through a beam expander and was focused by a microscope objective (Olympus 10x, NA = 0.3) into the sample. The power of the incident light is controlled by a half-wave plate and a polarizing beam splitter. In the visible range, the writing energies were 16 J and 20 J corresponding to sample LiNbO_3_ with Λ=6.8 μm and sample quartz with Λ*=*41.6 μm for 1064 nm SHG experiment, respectively. In the ultraviolet range, the writing energies were 8J and 12 J corresponding to quartz samples with *L_a_= L_b_=*1.4 μm for 428 nm SHG experiment and *L_a_=L_b_=*2.1 μm for 484 nm SHG experiment, respectively. The size of the focal spot inside the sample was ~2 μm in the transverse direction and 15-25 μm in the axis direction with the scanning speed of 1 mm/s. According to the written program, the shutter blocked the beam after fabricating a grating and the sample was moved to another location where the process was repeated. In order to obtain a high conversion efficiency, several layers were fabricated along the depth direction.

**SHG experiment with APP samples**

The SHG experiment of visible range was performed in both ferroelectric LiNbO_3_ and non-ferroelectric quartz. A Nd:YAG laser (100 ns, 20 kHz) illuminated the as-prepared sample by a focusing lens (f=100 mm) and the SHG signal was collected by a power meter. The SHG experiments of UV/DUV ranges were performed in crystal quartz. The fundamental beam was provided by an [optical parametric oscillator](file:///K:\学生发表论文资料\shaomingchuan\AppData\Local\youdao\dict\Application\7.5.2.0\resultui\dict\javascript:;) (OPO, Opolette TM HE 355 II, Coherent), which can generate lasers at the wavelength of 355 nm and tunable wavelength from 410 nm to 2200 nm with repetition rates of 20 Hz. The pulse width of the laser at 355 nm is 10 ns, and that at the wavelengths from 400 to 1064 nm is about 12 ns. The beam propagated along the z axis of the crystal quartz and was polarized along the x direction to make use of the largest nonlinear coefficient *d_11_* in the frequency conversion process. After focused by a 100 mm lens, the fundamental beam was incident into the sample with the focal waist of 50 μm. A CaF_2_ prism was employed to eliminate the overlap of the fundamental and SHG lights with the Brewster angle of incidence. With a power meter we can measure the power of SHG light. To reduce the absorption of the deep ultraviolet light by oxygen in the air, a chest filled with nitrogen was applied in the SHG experiments at 177.3 nm.

1. **Theoretical analysis on the additional period phase concept for phase-matching**

During the typical collinear frequency doubling process, the electric field *E_2ω_*(z) of the SHG light is expressed as ^[1]^

$\frac{dE_{2\omega}(z)}{dz}=\frac{2i\omega}{cn_{2\omega}(z)}d_{\mathrm{eff}}(z)E_{\omega}^{2}(z)e^{-i\Delta\varphi}$ (S1)

where *E_ω_* (z) denotes the electric field of the fundamental field at the propagation length *z*; *ω* refers to the fundamental frequency; *c* represents the light velocity; *n_2ω_*(z) and *d_eff_* (z) denote the refractive indexes of the SHG light and effective nonlinear coefficient at the propagation length *z*; is the phase difference between the fundamental and SHG lights with the wavevectors *k_1_* and *k_2_*, respectively.

For the phase-matching condition, , which can be achieved by using the birefringence of anisotropic crystals. For the quasi-phase-matching condition,, where *G* denotes the reciprocal vector introduced by the Fourier expansion of the nonlinear coefficient *d_eff_* as $d_{eff}(z)\text{=}d_{eff}f(z)$,with *f*(z) is the Fourier series that can be met by periodically reversing the polarization of the ferroelectric domains corresponding to the sign of the *d_eff_*.

In fact, from the mathematical perspective, the periodic variation of the parameters can be expressed by the Fourier expansion, suggesting that the periodic variation of *n_2ω_*(z) or *d_eff_* (z) can introduce the reciprocal vector *G*. Nevertheless, from the perspective of nonlinear optics, when , the energy will transfer from the SHG to the fundamental light, and the SHG output will oscillate with a phase difference period of 2π, as shown in **Fig. 1**. Accordingly, the additional phase difference (APP) concept can be proposed. It is expressed as follows: when the phase difference  reaches π, the SHG process will be blocked, and an APP with the value of π or -π will be added; after adding the APP, the phase difference in a certain period will be or 0, here *m* is an integer.

From the experimental perspective, the APP can be achieved by periodically processing, which undermines the translational symmetry of the nonlinear crystals. In the unbroken regions, the frequency conversion can reach its maximum when . There exists no continuous SHG effects in the processing regions, whereas the dispersion of the refractive indices exists with wavevectors *k_1_* and *k_2_*, thereby generating an APP . When the fundamental and SHG lights propagate for a certain period, the phase difference between the fundamental and SHG lights should be , which can reach 0 or 2mπ to avoid the transferring of energy from the SHG light to the fundamental light, where *m* is an integer, as shown in **Fig. 1d.**

From the perspective of mathematics, the physical parameters in Eq. (S1) can be transformed as

$\frac{d_{eff}(z)}{n_{2\omega}(z)}=\frac{d_{eff}}{n_{2\omega}}f(z)$ (S2)

With the period of Λ, the Fourier series *f*(z) is written as

$f(z)=c_{0}+\sum_{\begin{aligned} &m=-\infty\\ &m\neq0 \end{aligned}}^{+\infty} c_{m}e^{iG_{m}z}$ (S3)

With $c_{m}=\left\{ \begin{matrix} D & m=0 \\ \frac{1}{m\pi}\sin(Dm\pi) & m\neq0 \end{matrix} \right.$ and reciprocal vector $G_{m}=\frac{2m\pi}{\Lambda}$, where $D=\frac{l_{a}}{l_{b}+l_{a}}=\frac{l_{a}}{\Lambda}$ denotes the duty ratio; *l_a_* refers to the length of the periodic regions for the effective nonlinear interaction between the fundamental and SHG lights; *l_b_* represents the length of periodic regions where phase-difference is added; the conversion is blocked from the SHG to the fundamental light.

Based on Eq. (S2) and (S3), Eq. (S1) can be transformed to

$\frac{dE_{2\omega}(z)}{dz}=\frac{2i\omega}{cn_{2\omega}}d_{\mathrm{eff}}E_{\omega}^{2}(z)(c_{0}+\sum_{\begin{aligned} &m=-\infty\\ &m\neq0 \end{aligned}}^{+\infty} c_{m}e^{iG_{m}z})e^{-i\Delta\varphi}$ (S4)

If , i.e. $k_{2}-2k_{1}\text{= }G_{m}$, the phase matching can be satisfied, and the reciprocal vector *G_m_* will predominate in the frequency conversion process.

**3. Measurement of refractive index under different incident femtosecond laser energy intensities**


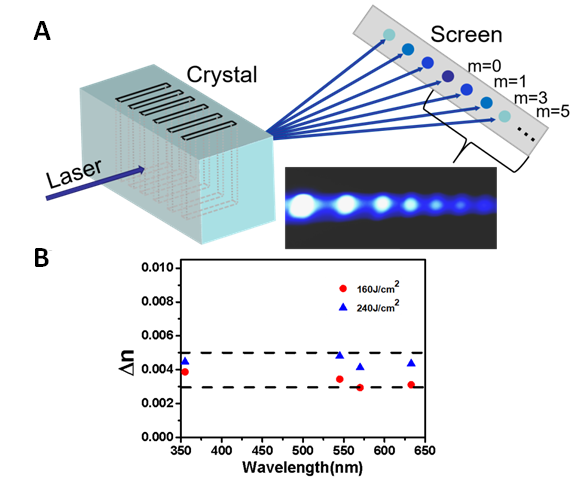


**Figure S1.** **Characterization of refractive index change of quartz induced by femtosecond laser.** (**A**) The schematics for the measurement of the refractive index with D (duty ratio) =1/2. (**B**) The dispersion of the change of the refractive index written with different energy intensities.

When the light transmits a bulk grating, the diffraction will occur, and in accordance with the Bragg diffraction theory, the difference of the refractive index in the grating can be calculated by measuring the diffracted light power ^[2, 3]^. When a light beam passes through a grating, its energy can be diffracted to different energy levels. The resulting phase difference can be expressed as

(S5)

where ∆n denotes the refractive index change; h is the grating’s thickness. For rectangle waveguide gratings, the equation for the efficiency of each diffraction order is written as

$\left\{ \begin{matrix} \eta_{0}=1-2D(1-D)(1-cos\Delta\varphi) \\ \eta_{m}>0=\frac{1}{m^{2}\pi^{2}}(1-cos2m\pi D)(1-cos\Delta\varphi) \end{matrix} \right.$ (S6)

where η_0_ and η_m_>0 are the diffraction efficiencies of different orders, respectively, D is the duty ratio of grating. By measuring the ratios of various diffraction orders, the change of refractive index with different energy intensities can be calculated.

The schematics for the measurement of the refractive index are given in **Fig. S1**. The laser at different wavelengths propagated along the Y direction of the APP crystal, and the diffraction series were then measured with a power meter. Based on the calculation of Eq. (S6), the change of the refractive index by laser writing can be obtained with the results shown in **Fig. S1B**, showing that the change of refractive index varies in the range from 0.003 to 0.005.

1. **Dispersion equation of quartz and LiNbO_3_.**

Notably, the dispersion equation of quartz is adopted in ref [4].

$$n_{o,e}^{2}=A_{o,e}+\frac{B_{o,e}\lambda^{2}}{\lambda^{2}-C_{o,e}}+\frac{D_{o,e}\lambda^{2}}{\lambda^{2}-F_{o,e}}$$

|  | A | B | C (10^-2^) | D | F |
| --- | --- | --- | --- | --- | --- |
| n_o_ | 1.28604141 | 1.07044083 | 1.00585997 | 1.10202242 | 100 |
| n_e_ | 1.28851804 | 1.09509924 | 1.02101864 | 1.15662475 | 100 |

**Figure S2** shows wavelength dependence of required period for 1st-order APP SHG from 150 nm to 600 nm.


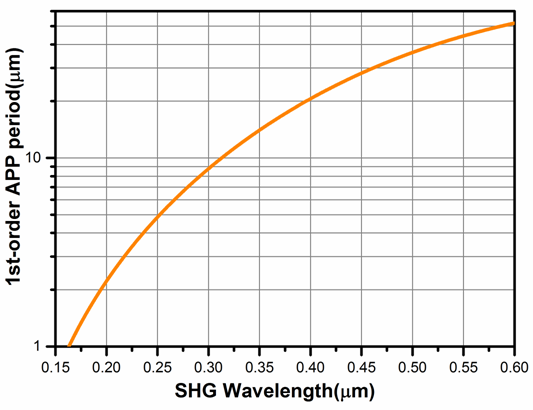


**Figure S2**. Wavelength dependence of required period for 1st-order APP SHG.

The dispersion equation of LiNbO_3_ is adopted in ref [5].

$$n_{o,e}^{2}={1+A}_{o,e}+\frac{A_{o,e}\lambda^{2}}{\lambda^{2}-B_{o,e}}+\frac{C_{o,e}\lambda^{2}}{\lambda^{2}-D_{o,e}}+\frac{E_{o,e}\lambda^{2}}{\lambda^{2}-F_{o,e}}$$

|  | A | B | C | D | E | F |
| --- | --- | --- | --- | --- | --- | --- |
| n_o_ | 2.6734 | 0.01764 | 1.2290 | 0.05914 | 12.614 | 474.6 |
| n_e_ | 2.9804 | 0.02047 | 0.5981 | 0.0666 | 8.9543 | 416.08 |

1. **Characterization of the anisotropy of the processed regions**

The crystal quartz SiO_2_ is a uniaxial crystal with the point group of 32. When polarization direction of the incident light is parallel to the XZ or YZ face, the anisotropic properties can be obviously observed under a polarizing microscope (**Fig. S3A**). During the measurement, the polarized light propagated along the Y direction. After the crystal, the light propagates through a polarizer with the polarization direction vertical to that of the first polarizer. By rotating the crystal around the Y direction, the patterns were observed, and the anisotropy of the crystal was checked as shown in **Fig. S3B** where the broken regions stay dark regardless of the polarization of the incident light proving that the broken regions are isotropic.


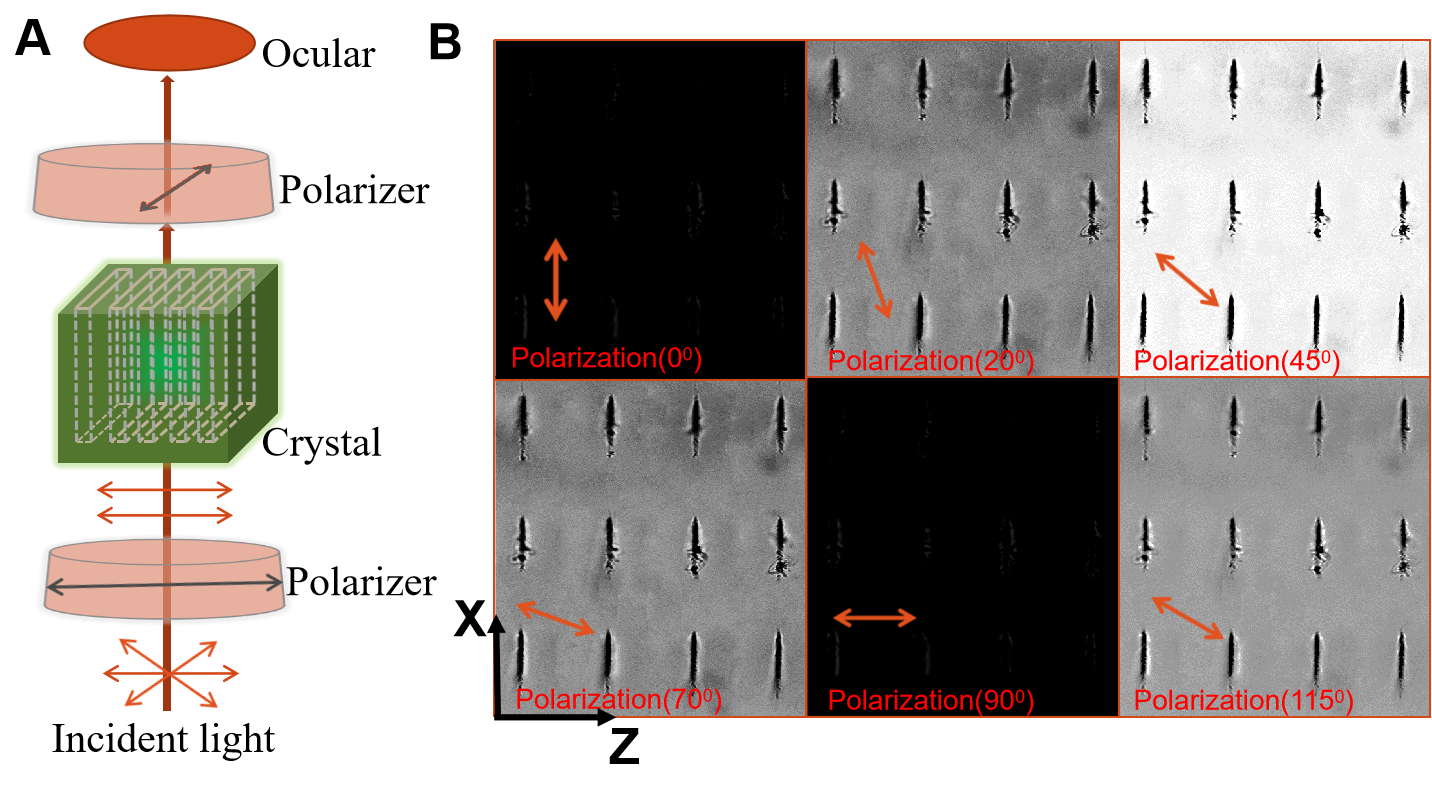


**Figure S3. The measurement of the anisotropy of the APP SiO_2_ sample.** (A) Schematics of the polarizing microscope. (B) Observed patterns of the APP SiO_2_ sample under a polarizing microscope.

1. **Experimental configuration for the second harmonic generation of the APP SiO_2_**

The second-order nonlinear susceptibility tensor of the crystal quartz is expressed as (*11, 27*)

$\chi^{\text{(2)}}\text{=}\left(\begin{matrix} \chi_{11} & -\chi_{11} & 0 & \chi_{14} & 0 & 0 \\ 0 & 0 & 0 & 0 & -\chi_{14} & -\chi_{11} \\ 0 & 0 & 0 & 0 & 0 & 0 \end{matrix}\right)$ (S7)

Given Kleimann symmetry, the nonlinear susceptibility $\chi_{14}=-\chi_{14}=0$, and the susceptibility is$\chi_{ij}=2d_{ij}$, with nonlinear coefficient *d_ij_*. The nonlinear polarization along X ($P_{x}^{2\omega}$) and Y ($P_{y}^{2\omega}$) directions is written as

$\begin{matrix} P_{x}^{2\omega}=2\varepsilon_{0}[d_{11}(E_{x}^{\omega})^{2}-d_{11}(E_{y}^{\omega})^{2}] \\ P_{y}^{2\omega}=-4\varepsilon_{0}d_{11}E_{x}^{\omega}E_{y}^{\omega} \end{matrix}$ (S8)

where $\varepsilon_{0}$ denotes the vacuum dielectric constant; $E_{x}^{\omega}$ and $E_{y}^{\omega}$ represent the electric field component of fundamental light along X and Y direction, respectively.

An [optical parametric oscillator](C:/Users/shaomingchuan/AppData/Local/youdao/dict/Application/7.5.2.0/resultui/dict/javascript:;) (OPO, Opolette ^TM^ HE 355 II) served as the pump source, which can generate lasers at the wavelength of 355 nm and tunable wavelengths from 410 nm to 2200 nm with repetition rates of 20 Hz. In crystal quartz, the SHG with the polarization of fundamental laser parallel and about 45° to Y direction was also investigated, and the results are consistent with that of Eq. (S8). Based on Eq. (S8), the phase matching conditions are unchanged by the optical active properties and can be estimated by Eq. (S1), since the refractive indices of SiO_2_ along X and Y directions are equal.

1. **Broadband second harmonic generation of the APP SiO_2_**


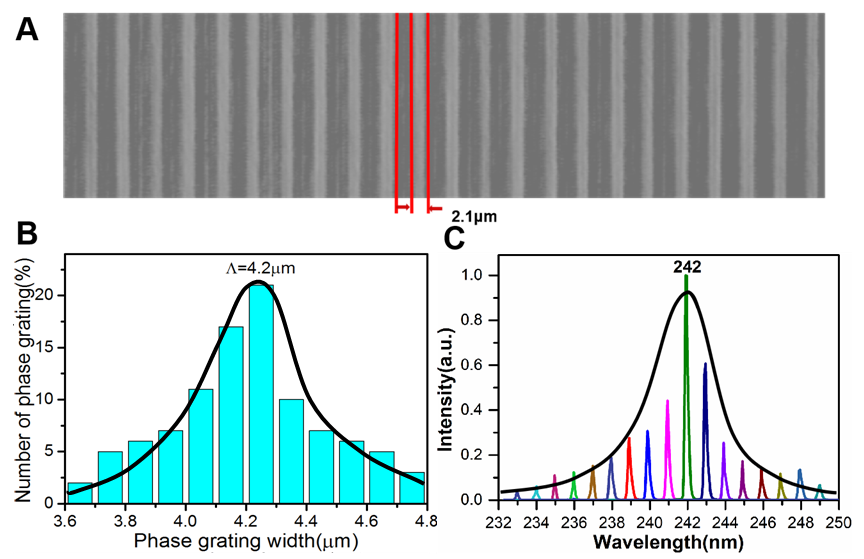


**Figure S4.** **Broadband SHG of the APP SiO_2_ sample with *l_a_=l _b_=* 2.1 μm.** (A) The pattern of the sample observed along Y direction with Λ= *l_a_ + l _b_*=4.2 μm. (B) The distribution of periodic grating widths. (C) The relative intensities of broadband second harmonic generation.


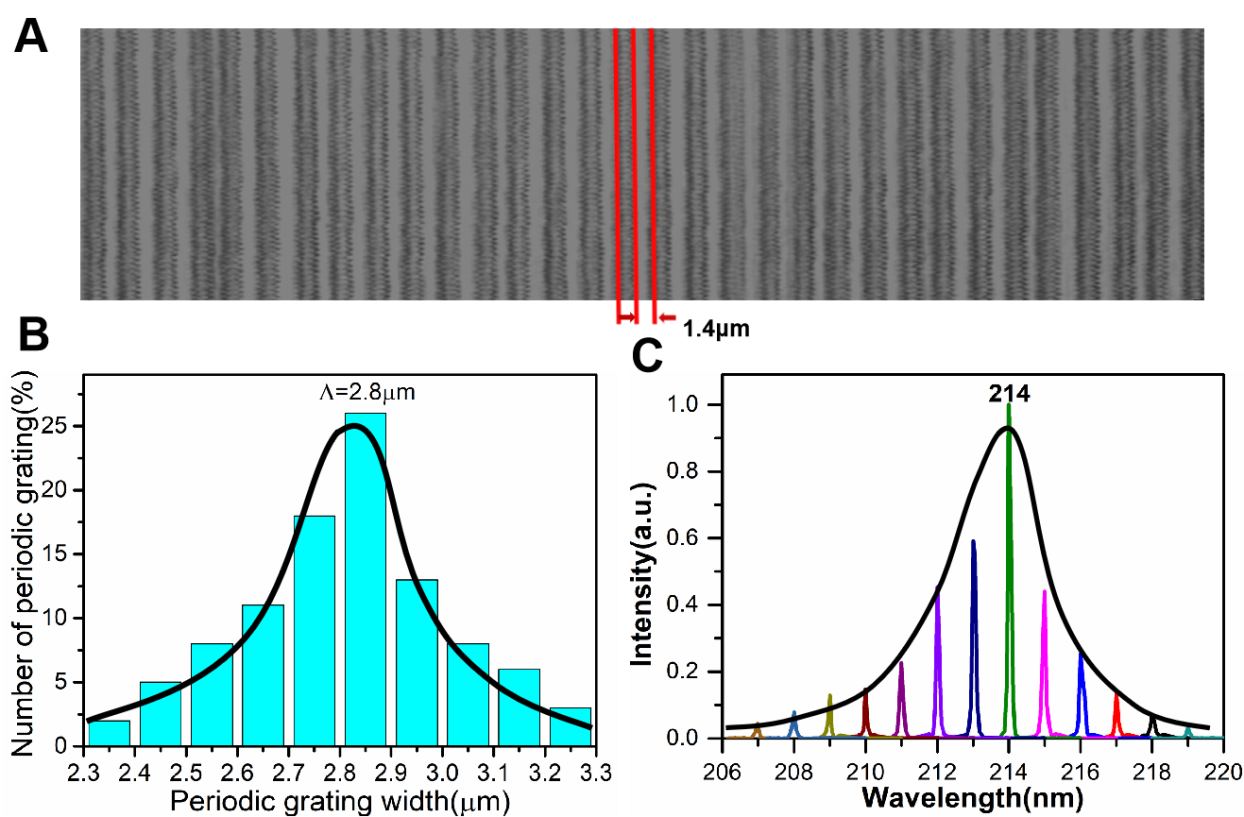


**Figure S5.** **Broadband SHG of the APP SiO_2_ sample with *l_a_= l _b_=* 1.4 μm.** (A) The pattern of the sample with Λ= *l_a_ + l _b_*=2.8 μm. (B) The distribution of periodic grating widths. (C) The relative intensities of broadband second harmonic generation.

Under the employed laser writing technique, the minimum focused diameter of femtosecond laser is about 2 μm. As shown in **Fig. S4**, the designed period is 4.2 μm for the 3^th^ order APP SiO_2_ sample. With a microscope, the period length was observed ranging from 3.6 to 4.8 μm, corresponding to the reciprocal vectors from 1.31 to 1.75 μm^-1^, which means that the uncertainty is about ±0.6 μm (±14.3%). While for the 2^th^ order APP SiO_2_ sample with the designed period of 2.8 μm (**Fig. S5**), the period length was observed ranging from 2.3 to 3.3 μm with the uncertainty of ±0.5 μm (±17.9%), corresponding to the reciprocal vectors from 1.90 to 2.73 μm^-1^. With the OPO, the relative output power was checked, which was also recorded with an optical spectrum analyzer (Maya 2000 PRO, Ocean Optics Inc.), as shown in **Fig. S4C** and **S5C**. These figures reveal that the SHG peaks located at 242 and 214 nm, with broadband ranges from 226 nm to 252 nm and 205 nm to 221 nm, respectively, which are consistent with the provided reciprocal vectors.

1. **The basic properties of DUV APP candidates for 177.3 nm generation**

**Table S1.** A brief list for basic properties of DUV nonlinear crystals

| Crystal | Space group | UV cutoff  (nm) | Max d_ij_  (pm/V) | Ferroelectric | Spontaneous  domain | Deliquescence | Commercial  crystal |
| --- | --- | --- | --- | --- | --- | --- | --- |
| KBBF | R32 | 147 | 0.47 | No | No | No | Yes |
| Quartz | P3_1_21 | 146 | 0.3 | No | No | No | Yes |
| LiB_3_O_5_ | Pna2_1_ | 155 | 0.78 | No | No | Yes | Yes |
| BPO_4_ | I$\overline{4}$ | 134 | 0.76 | No | No | No | No |
| SrB_4_O_7_ | Pmn2_1_ | ~130 | 0.62 | No | Yes | No | No |
| Mg_3_B_7_O_13_Cl | Pca2_1_ | 155 | 0.38 | Yes | Yes | No | No |
| SrAlF_5_ | I4 | 155 | 0.3 | Yes | Yes | No | No |
| BaMgF_4_ | Cmc2_1_ | 125 | 0.12 | Yes | Yes | No | No |
| SrMgF_4_ | P2_1_ | 122 | 0.05 | Yes | Yes | No | No |

**References**

1. Boyd, R. W. Nonlinear Optics. *Academic Press* (2008).

2. Wei, D. Z. *et al*. Experimental demonstration of a three-dimensional lithium niobate nonlinear photonic crystal. *Nature Photonics* **12**, 596-600 (2018).

3. Davis, K. M. *et al*. Writing waveguides in glass with a femtosecond laser. *Optics Letters* **21**, 1729-1731 (1996).

4. Ghosh, G. Dispersion-equation coefficients for the refractive index and birefringence of calcite and quartz crystals. *Optics Communications* **163**, 95-102 (1999).

5. Zelmon, D. E. , Small, D. L. & Jundt, D. Infrared Corrected Sellmeier Coefficients for Congruently Grown Lithium Niobate and 5 mol. % Magnesium Oxide-doped Lithium Niobate. *Journal of the Optical Society of America B* **14**, 3319-3322 (1997).
